# Supplementary material for: Ability of known colorectal cancer susceptibility SNPs to predict colorectal cancer risk: A cohort study within the UK Biobank
Source: PLoS One. 2021 Sep 15;16(9):e0251469. doi: 10.1371/journal.pone.0251469 (PMC8443076; doi:10.1371/journal.pone.0251469)
Supplement: S1 Table — (PDF) [file pone.0251469.s001.pdf]

S1 Table: SNPs used to generate PRS

rsID	Locus	Effect allele	Reference allele	EAF	Odds ratio
rs6669796	1q25.3	G	C	0.54	1.05
rs6687758	1q41	G	A	0.20	1.09
rs72647484	1p36.2	T	C	0.91	1.21
rs11903757	2q32.3	C	T	0.36	1.06
rs10936599	3q26.2	C	T	0.75	1.08
rs35360328	3p22.1	A	T	0.16	1.14
rs812481	3p14.1	G	C	0.58	1.09
rs11100443	4q32.2	T	C	0.09	1.53
rs3987	4q26	G	A	0.44	1.36
rs647161	5q31.1	A	C	0.67	1.11
rs1321311	6p21.31	A	C	0.23	1.10
rs16892766	8q23.3	C	A	0.07	1.25
rs6983267	8q24.21	G	T	0.52	1.21
rs719725	9q24	C	A	0.37	1.19
rs10795668	10p14	G	A	0.67	1.12
rs10904850	10p13	G	A	0.68	1.14
rs11190164	10q24.2	G	A	0.29	1.09
rs12241008	10q25	C	T	0.09	1.13
rs704017	10q22.3	G	A	0.57	1.06
rs174537	11q12.2	T	G	0.57	1.40
rs3802842	11q23.1	C	A	0.29	1.11
rs3824999	11q13.4	G	T	0.50	1.08
rs10774214	12p13.32	T	C	0.38	1.09
rs11169552	12q13.13	C	T	0.72	1.09
rs3184504	12q24.12	C	T	0.53	1.09
rs3217810	12p13.32	T	C	0.16	1.20
rs3217901	12p13.32	G	A	0.41	1.10
rs59336	12q24.21	T	A	0.48	1.09
rs7136702	12q13.13	T	C	0.35	1.06
rs73208120	12q24.22	G	T	0.11	1.16
rs1957636	14q22.2	T	C	0.40	1.08
rs4444235	14q22.2	C	T	0.46	1.11

rs11632715	15q13.3	A	G	0.47	1.12
rs16969681	15q13.3	T	C	0.09	1.18
rs16941835	16q22.1	C	G	0.21	1.15
rs9929218	16q22.1	G	A	0.71	1.10
rs744166	17q21	G	A	0.42	1.27
rs4939827	18q21.1	T	C	0.52	1.18
rs10411210	19q13.11	C	T	0.90	1.15
rs1800469	19q13.2	G	A	0.49	1.16
rs11204472	20q13.33	A	G	0.68	1.08
rs2423279	20p12.3	C	T	0.30	1.14
rs4813802	20p12.3	G	T	0.36	1.09
rs6066825	20q13.1	A	G	0.64	1.09
rs961253	20p12.3	A	C	0.36	1.12

Abbreviations: EAF, effect allele frequency
