# Supplementary material for: Ability of known colorectal cancer susceptibility SNPs to predict colorectal cancer risk: A cohort study within the UK Biobank
Source: PLoS One. 2021 Sep 15;16(9):e0251469. doi: 10.1371/journal.pone.0251469 (PMC8443076; doi:10.1371/journal.pone.0251469)
Supplement: S2 Table — (PDF) [file pone.0251469.s002.pdf]

**S2 Table: Standardised incidence ratios (SIR) by age group for full lifetime and 10-year risk (combined model)**

| | Observed | Expected | SIR | 95% CI |
|---|---|---|---|---|
| **Full lifetime risk: 40–49 years** | | | | |
| Bottom 10% | 5 | 6.67 | 0.75 | 0.31–1.80 |
| Decile 2 | 2 | 6.81 | 0.29 | 0.07–1.17 |
| Decile 3 | 9 | 6.95 | 1.29 | 0.67–2.48 |
| Decile 4 | 6 | 7.03 | 0.85 | 0.38–1.89 |
| Decile 5 | 7 | 6.96 | 1.00 | 0.48–2.11 |
| Decile 6 | 14 | 7.15 | 1.95 | 1.15–3.30 |
| Decile 7 | 5 | 7.04 | 0.71 | 0.29–1.70 |
| Decile 8 | 9 | 7.06 | 1.27 | 0.66–2.45 |
| Decile 9 | 13 | 7.08 | 1.83 | 1.06–3.16 |
| Top 10% | 12 | 6.96 | 1.72 | 0.98–3.03 |
| **Full lifetime risk: 50–59 years** | | | | |
| Bottom 10% | 32 | 42.32 | 0.75 | 0.53–1.07 |
| Decile 2 | 27 | 43.94 | 0.6145 | 0.42–0.89 |
| Decile 3 | 40 | 44.81 | 0.8927 | 0.65–1.21 |
| Decile 4 | 63 | 45.42 | 1.3871 | 1.08–1.77 |
| Decile 5 | 46 | 47.32 | 0.972 | 0.72–1.29 |
| Decile 6 | 49 | 46.65 | 1.0503 | 0.79–1.39 |
| Decile 7 | 54 | 47.73 | 1.1314 | 0.86–1.47 |
| Decile 8 | 47 | 49.22 | 0.9548 | 0.71–1.27 |
| Decile 9 | 62 | 48.89 | 1.2681 | 0.98–1.62 |
| Top 10% | 80 | 50.61 | 1.58 | 1.27–1.96 |
| **Full lifetime risk: 60–69 years** | | | | |
| Bottom 10% | 106 | 147.22 | 0.72 | 0.59–0.87 |
| Decile 2 | 98 | 161.11 | 0.60 | 0.5–0.74 |
| Decile 3 | 103 | 172.62 | 0.59 | 0.49–0.72 |
| Decile 4 | 158 | 181.87 | 0.86 | 0.74–1.01 |
| Decile 5 | 155 | 186.32 | 0.83 | 0.71–0.97 |

| | Observed | Expected | SIR | 95% CI |
|---|---|---|---|---|
| Decile 6 | 163 | 193.28 | 0.84 | 0.72–0.98 |
| Decile 7 | 176 | 199.1 | 0.88 | 0.76–1.02 |
| Decile 8 | 204 | 202.27 | 1.00 | 0.88–1.15 |
| Decile 9 | 217 | 210.48 | 1.03 | 0.90–1.17 |
| Top 10% | 285 | 218.90 | 1.30 | 1.16–1.46 |
| **Full lifetime risk: 70–75 years** | | | | |
| Bottom 10% | 35 | 63.67 | 0.55 | 0.39–0.76 |
| Decile 2 | 61 | 72.08 | 0.84 | 0.65–1.08 |
| Decile 3 | 73 | 76.38 | 0.95 | 0.76–1.20 |
| Decile 4 | 74 | 79.89 | 0.92 | 0.73–1.16 |
| Decile 5 | 63 | 83.17 | 0.75 | 0.59–0.97 |
| Decile 6 | 64 | 87.03 | 0.73 | 0.57–0.94 |
| Decile 7 | 61 | 89.5 | 0.68 | 0.53–0.87 |
| Decile 8 | 92 | 92.89 | 0.99 | 0.80–1.21 |
| Decile 9 | 101 | 96.68 | 1.04 | 0.86–1.27 |
| Top 10% | 121 | 102.09 | 1.18 | 0.99–1.41 |
| **10-year risk: 40–49 years** | | | | |
| Bottom 10% | 26 | 36.63 | 0.71 | 0.48–1.04 |
| Decile 2 | 33 | 18.17 | 1.81 | 1.29–2.55 |
| Decile 3 | 14 | 7.95 | 1.76 | 1.04–2.97 |
| Decile 4 | 6 | 3.54 | 1.69 | 0.76–3.77 |
| Decile 5 | 0 | 1.7 | 0 | – |
| Decile 6 | 2 | 0.86 | 2.33 | 0.58–9.32 |
| Decile 7 | 0 | 0.47 | 0 | – |
| Decile 8 | 1 | 0.25 | 4.07 | 0.57–28.93 |
| Decile 9 | 0 | 0.13 | 0 | – |
| Top 10% | 0 | 0.04 | 0 | – |
| **10-year risk: 50–59 years** | | | | |
| Bottom 10% | 23 | 35.10 | 0.65 | 0.43–0.98 |
| Decile 2 | 63 | 80.37 | 0.78 | 0.61–1.00 |
| Decile 3 | 88 | 85.03 | 1.03 | 0.84–1.27 |
| Decile 4 | 84 | 72.01 | 1.16 | 0.94–1.44 |

| | Observed | Expected | SIR | 95% CI |
|---|---|---|---|---|
| Decile 5 | 56 | 57.57 | 0.97 | 0.74–1.26 |
| Decile 6 | 56 | 44.27 | 1.26 | 0.97–1.64 |
| Decile 7 | 40 | 34.49 | 1.16 | 0.85–1.58 |
| Decile 8 | 44 | 27.02 | 1.62 | 1.21–2.18 |
| Decile 9 | 29 | 19.51 | 1.48 | 1.03–2.13 |
| Top 10% | 17 | 11.54 | 1.47 | 0.91–2.37 |
| **10-year risk: 60–69 years** | | | | |
| Bottom 10% | 0 | 1.14 | 0 | – |
| Decile 2 | 19 | 27.81 | 0.68 | 0.43–1.07 |
| Decile 3 | 74 | 90.6 | 0.81 | 0.65–1.02 |
| Decile 4 | 90 | 153.13 | 0.58 | 0.47–0.72 |
| Decile 5 | 142 | 200.97 | 0.70 | 0.60–0.83 |
| Decile 6 | 196 | 239.53 | 0.81 | 0.71–0.94 |
| Decile 7 | 199 | 266.59 | 0.74 | 0.65–0.85 |
| Decile 8 | 258 | 284.76 | 0.90 | 0.80–1.02 |
| Decile 9 | 311 | 298.77 | 1.04 | 0.93–1.16 |
| Top 10% | 376 | 309.88 | 1.21 | 1.09–1.34 |
| **10-year risk: 70–75 years** | | | | |
| Bottom 10% | – | – | – | – |
| Decile 2 | 2 | 0.41 | 4.82 | 1.20–19.3038 |
| Decile 3 | 4 | 7.78 | 0.51 | 0.19–1.37 |
| Decile 4 | 14 | 26.49 | 0.52 | 0.31–0.89 |
| Decile 5 | 38 | 52.34 | 0.72 | 0.52–0.99 |
| Decile 6 | 62 | 82.54 | 0.75 | 0.58–0.96 |
| Decile 7 | 98 | 111.26 | 0.88 | 0.72–1.07 |
| Decile 8 | 117 | 143.85 | 0.81 | 0.67–0.97 |
| Decile 9 | 146 | 180.03 | 0.81 | 0.69–0.95 |
| Top 10% | 264 | 238.69 | 1.10 | 0.98–1.24 |

Abbreviation: SIR, standardised incidence ratio
